# Multicenter evaluation of the QIAstat Respiratory Panel—A new rapid highly multiplexed PCR based assay for diagnosis of acute respiratory tract infections

**DOI:** 10.1371/journal.pone.0230183

**Published:** 2020-03-12

**Authors:** Marijo Parčina, Uffe Vest Schneider, Benoit Visseaux, Robert Jozić, Irene Hannet, Jan Gorm Lisby

**Affiliations:** 1 Institute of Medical Microbiology, Immunology and Parasitology, University Hospital Bonn, Bonn, Germany; 2 Department of Clinical Microbiology, Copenhagen University Hospital Hvidovre, Hvidovre, Denmark; 3 IAME, UMR 1137, INSERM, Université Paris Diderot, Sorbonne Paris Cité, AP-HP, Laboratoire de Virologie, Hôpital Bichat, AP-HP, Paris, France; 4 STAT-Dx Life (Qiagen) Parc Científic, Barcelona, Spain; Hong Kong Institute for the Humanities and Social Sciences, HONG KONG

## Abstract

Acute respiratory tract infections (ARTI), including the common cold, pharyngitis, sinusitis, otitis media, bronchiolitis and pneumonia are the most common diagnoses among patients seeking medical care in western countries, and account for most antibiotic prescriptions. While a confirmed and fast ARTI diagnosis is key for antibiotic prescribing, empiric antimicrobial treatment remains common, because viral symptoms are often clinically similar and difficult to distinguish from those caused by bacteria. As a result, inappropriate antibiotic prescriptions are high and in certain settings likely higher than the commonly estimated 30%. The QIAstat Respiratory Panel^®^ assay (QIAstat RP) is a multiplexed in vitro diagnostics test for the rapid simultaneous detection of 21 pathogens directly from respiratory samples, including human mastadenovirus A-G, primate bocaparvovirus 1+2, human coronavirus (HKU1, NL63, OC43, 229E), human metapneumovirus A/B, rhinovirus/enterovirus, influenza A virus (no subtype, subtype H1, H1N1/2009, H3), influenza B virus, human respirovirus 1+3, human orthorubulavirus 2+4, human orthopneumovirus, *Bordetella pertussis*, *Chlamydia pneumoniae*, *Mycoplasma pneumoniae* and *Legionella pneumophila*. We describe the first multicenter study of 445 respiratory samples, collected through the 2016–2017 and 2018 respiratory seasons, with performance compared against BioFire FilmArray RP v1.7 and discrepancy testing by Seegene Allplex RP. The QIAstat RP demonstrated a positive percentage of agreement of 98.0% (95% CI: 96.0–99.1%) and a negative percentage agreement of 99.8% (95% CI: 99.6–99.9%). With use of this comprehensive and rapid test, improved patient outcomes and antimicrobial stewardship may potentially be achieved.

## Introduction

Acute respiratory tract infections (ARTI) are a leading cause of morbidity and mortality worldwide and a frequent reason for seeking medical care [1]. Most pathogens have predilection for either the upper or lower respiratory tract but may move into the other compartment. The etiology is diverse, led by a high viral heterogeneity and also comprises bacteria [2,3]. The health-related burden of acute respiratory tract infections is significant in defined populations like the very young, the elderly, the chronically ill with underlying comorbidities (e.g., heart or lung disease, diabetes), the malnourished and the immunocompromised (e.g., transplant recipients, HIV-infected individuals) [4–9].

Certain pathogens, including influenza A/B virus, human orthopneumovirus (also known as respiratory syncytial virus) and *Bordetella pertussis*, conventionally require contact isolation of hospitalized patients when detected, to avoid further institutional spread [10]. Their fast detection and identification are essential for infection control effectiveness.

Acute respiratory signs and symptoms are seldom‬‬ specific for a single pathogen, and detection strategies‬‬ that allow for multiple agents to be simultaneously detected, may have a significant impact on infectious‬‬‬‬ disease management [11–13]. For the patient, the ability to rapidly and accurately rule in /out a respiratory pathogen supports optimized overall care (e.g. hospital admission or not) and therapy selections, e.g. terminating unnecessary antimicrobials and targeted use of antiviral agents [14–18]. From a public health perspective, syndromic respiratory infection testing has shown to be a useful tool for seasonal and sporadic outbreak surveillance and preparedness [19], supporting the effective management of health care resources [20].

Thus, as respiratory viruses and their potential role are increasingly acknowledged, the use of multiplex PCR testing is recommended since December 2018 by the Infectious Diseases Society of America in all immunocompromised populations presenting with ARTI symptoms and also suggested in other populations if their results are taken into account to improve patients management [21].

This study describes the first multicenter evaluation of the clinical performance characteristics of the QIAstat Respiratory Panel (QIAstat RP, Qiagen, Hilden, Germany) on respiratory samples, compared to FilmArray Respiratory Panel Assay v1.7 (FilmArray RP, bioMérieux, Marcy l’Etoile, France) with discrepancy testing performed by Allplex™ Respiratory Panel (Allplex RP, Seegene, Seoul, South Korea) in two geographic settings in Europe.

## Material and methods

### Study design and sites

The study was designed as an observational, prospective-retrospective study aiming to test all QIAstat RP pathogens from fresh or frozen samples. The study was performed at two clinical sites located in Denmark and Germany.

The Department of Clinical Microbiology, University Hospital of Hvidovre (Copenhagen, Denmark) tested 228 respiratory samples collected in universal transport media (UTM). Samples were prospectively collected during an enrolment period between February 5th and February 22^nd^, 2018.

The Department of Clinical Microbiology, University hospital of Bonn, Germany tested 217 protocol compliant retrospective clinical samples between January 24th and March 21th 2018. Samples had been collected during the 2016–2017 influenza season (December 2016–May 2017) as flocked swab samples in UTM, and residual sample had been stored at -80°C.

All samples were tested by the QIAstat RP (Qiagen, Hilden Germany) and the FilmArray RP v1.7 (bioMérieux, Marcy l´ Etoile, France) that served as the reference method. Discrepancies between the two test methods for both clinical sites were resolved by Allplex RP (Seegene, Seoul, South Korea) at the clinical site in Bonn.

Testing at both clinical sites was performed by trained laboratory personnel, who had shown proficiency with both methods.

### Clinical samples

Residual respiratory samples as nasopharyngeal flocked swabs in UTM (Copan, Brescia, Italy) received for routine bacterial and viral testing from 445 patients were included in the study. Samples were enrolled consecutively according to instrument capacity. Clinical samples were included from two study sites after meeting the following inclusion criteria: Respiratory samples were collected utilizing flocked swab (FLOQSwabs™,Copan, Brescia, Italy) placed in UTM and were sent for viral and/or bacterial testing. The residual volume of all included samples was above 600 μL. All samples were tested by the QIAstat RP and the Filmarray RP within 4 days of collection when stored at 2–8°C, and within 90 minutes after thawing when stored at -80 C.

The samples were anonymized and assigned a study number linked to patient demographic information, including age, sex and hospitalization status (general practice, hospitalized, pediatric hospital/ward, intensive care unit). The samples had been collected under an institutional review board (IRB)-approved protocol, which included a waiver of informed consent for the use of residual anonymized samples. The protocol was approved by Ethikkommission an der Medizinischen Fakultät der Rheinischen Friedrich-Wilhelms-Universität Bonn under the approval 052/18 and by De Videnskabsetiske Komiteer Center for Sundhed, Region Hovedstaden under the approval H-18001793.

### QIAstat Dx system and RP cartridge

The QIAstat Dx system is a new highly multiplexed platform for integrated nucleic acid extraction and multiplex, RT-real-time PCR detection. The QIAstat RP (Catalogue number 691211) is designed to run on the QIAstat DiagCORE Analyzer testing respiratory samples from individuals with signs and symptoms of an acute respiratory tract infection. Each QIAstat RP cartridge contains a full process Internal Control (IC), which is a MS2 bacteriophage that is included in dried format and gets automatically rehydrated upon sample loading. The IC verifies all steps of the analysis process during testing, including sample resuspension/homogenization, lysis, nucleic acid purification, reverse transcription, and PCR. The cartridge has two distinct loading ports and can be inoculated directly with a dry swab or with transport medium. In brief, samples were homogenized by vigorous inversion of the vial and 300 μL UTM was transferred into the assay cartridge using the provided transfer pipette. The inoculated cartridge was placed on the instrument. The test started automatically and ran for 69 minutes. Upon completing the test and ejecting the cartridge, the analyzer interpreted results and displayed a test summary. The analyzer will also report errors that may occur during processing. Amplification curves and cycle threshold (Ct) values can be viewed for detected pathogens and for the IC. A report can be printed or exported to an external USB storage device. The analyzer can be bidirectionally connected to laboratory information systems.

The QIAStat Dx system consists of one to four Analytical modules (Catalogue number 9002814) plus one Analyzer Module (Catalogue number 9002824) that allows for time independent parallel testing of one sample per module.

The QIAstat RP detects 17 viral and four bacterial pathogens including *human* mastadenovirus A-G (formerly adenovirus), primate bocaparvovirus 1+2 (formerly bocavirus), human coronavirus (differentiating HKU1, NL63, OC43 or 229E), human metapneumovirus A/B (hMPV), rhinovirus/enterovirus, influenza A virus (as no subtype, subtype H1, H1N1/2009 or H3), influenza B virus, human respirovirus 1 or 3, human orthorubulavirus 2 or 4 (formerly human parainfluenza virus type 1–4), human orthopneumovirus, *Mycoplasma pneumoniae*, *Legionella pneumophilia*, *Bordetella pertussis* and *Chlamydia pneumoniae*.

A QIAstat RP test was considered valid if the test was completed normally and the control passed. Results for influenza A virus were reported as influenza A virus subtype H3N2, influenza A virus subtype H1N1, or influenza A virus A pdm2009 for the pandemic 2009 influenza A strain—in case both the influenza A virus and the respective subtype/strain were detected. In case no subtype was detected, influenza A virus was reported as influenza A virus, no subtype.

This study was conducted with an investigational use only (IUO) version of the QIAstat RP. The final CE-marked QIAstat RP is identical to the IUO panel used in this study, with the exception that results for the *Chlamydia pneumoniae* target are not reported, pending regulatory clearance.

### FilmArray Respiratory Panel 1.7

The FilmArray RP is a multiplex sample-to-answer PCR panel that tests for 20 viral and bacterial pathogens on nasopharyngeal swabs in UTM at a time. A sample volume of 300 μL is required for the testing with approximately two minutes of hands-on-time in the set-up of the test.

The system consists of one to eight modules (BioFire FlimArray Torch, Salt Lake City, Utah, USA) that that allows for time independent parallel testing of one sample per module. The test runs for approximately 65 minutes. A report can be printed or exported to an external USB storage device. The analyzer can be bidirectionally connected to laboratory information systems.

The FilmArray RP v1.7 detects 17 viral and three bacterial pathogens including human mastadenovirus A-G, human coronavirus (differentiating HKU1, NL63, OC43 or 229E), human metapneumovirus A/B (hMPV), rhinovirus/enterovirus, influenza A virus (as no subtype, subtype H1, H1N1/2009 or H3), influenza B virus, human respirovirus 1 or 3, human orthorubulavirus 2 or 4, human orthopneumovirus, *Mycoplasma pneumoniae*, *Bordetella pertussis* and *Chlamydia pneumoniae*.

For human coronavirus HKU1 the FilmArray RP assay was accepted as correct as the pathogen is not included in the panel on the Allplex RP.

### Allplex Respiratory Panel

Samples that yielded discordant results between the QIAstat RP and the FilmArray RP were discrepancy tested by the Allplex RP assay.

The Allplex RP is a RT-PCR assay that tests for 19 viral and seven bacterial pathogens on nasopharyngeal swabs, nasopharyngeal aspirate or bronchoalveolar lavage at a time.

The Allplex RP runs on the Seegene workflow that consists of a module for nucleic acid extraction and PCR setup, a 96-well PCR thermocycler and a computer for interpretation and reporting with a total sample-to-answer time in less than one day.

The Allplex RP detects human mastadenovirus A-G, primate bocaparvovirus 1+2, human coronavirus (differentiating NL63, OC43 or 229E), human metapneumovirus A/B (hMPV), rhinovirus, enterovirus, influenza A virus (as no subtype, subtype H1, H1N1/2009 or H3), influenza B virus, human respirovirus 1 or 3, human orthorubulavirus 2 or 4, human orthopneumovirus A or B, *Mycoplasma pneumoniae*, *Bordetella pertussis*, *Bordetella parapertussis*, *Legionella pneumophila*, *Chlamydia pneumoniae*, *Streptococcus pneumoniae* and *Haemophilus influenzae*.

The Allplex RP assay was used as the comparator method for primate bocaparvovirus 1+2, as this analyte is not included in the FilmArray RP panel and the Allplex RP results were accepted as correct.

### Statistical analysis

Sensitivity and specificity are reported as the QIAstat RP could be compared to a combined result from the FilmArray RP and the Allplex RP. Results are reported as true positive (TP), false positive (FP), false negative (FN) and true negative (TN) results. Binomial two-sided 95% confidence intervals were calculated using the Wilson Score Method.

For human coronavirus HKU1 and primate bocaparvovius 1+2 results were compared by calculation of positive percent agreement (PPA) and negative percent agreement (NPA) as only results from the QIAstat RP and either the FilmArray RP or Allplex RP were available. Primate bocaparvovirus 1+2 is not included as a target in the FilmArray RP and the human coronavirus HKU1 is not included in the Allplex RP making it impossible to determine the sensitivity and specificity for these two targets. Accordingly, overall agreement is also reported as PPA and NPA as sensitivity and specificity was not determined for all targets.

The PPA represent how often a new test (NT) agrees with a non-reference standard (NRS) and is calculated as PPA = “NT and NRS positive” / (“NT and NRS positive” + “NT negative and NRS positive”), whereas the NPA is calculated as NPA = “NT and NRS negative” / (“NT and NRS negative” + “NT positive and NRS negative”).

## Results

### Patient demographics

445 patients were included in the study. Gender distribution was almost equal with 223 female patients and 222 male patients. 122 patients (27.4%) were younger than six years, 56 patients (12.6%) were between six and 21 years, 124 patients (27.9%) were between 22 and 49 years and 137 patients (30.8%) were 50 years or older. No age information was available for six patients (1.3%). The majority of samples, 307 (69.0%) were referred for testing by general practitioners. 106 samples (23.8%) were from hospitalized patients, 30 (6.8%) were from a pediatrics hospital/ward and two samples (0.4%) came from an intensive care unit. All patients had presented with signs and symptoms of an acute respiratory tract infection.

### Summary of QIAstat RP findings

The QIAstat RP detected one or more potential pathogens in 333 (74.8%) of the 445 tested samples (Table 1) for a total of 415 pathogens (Table 2).

**Table 1:** Total number of QIAstat RP positive samples according to number of detected pathogens per sample.

Pathogens detected by QIAstat RP	Number of samples	% of total samples (total = 445)
>0	333	74,8%
1	268	60,2%
2	53	11,9%
3	8	1,8%
4	3	0,7%
5	1	0,2%

**Table 2:** Number of QIAstat RP detected pathogens allocated by co-infection, hospitalization status and by age groups. Number of pediatric patients is outlined in superscript and number of intensive care unit patients is outlined in subscript.

Pathogen	Total number	Detected in co-infection	From hospitalized patient	<6 years	6–21 years	22–49 years	>49 years
Adenovirus	36	24	7^4^	21	8	6	1
Bocavirus	5	4	0	2	3	0	0
Coronavirus 229E	2	1	0	1	0	1	0
Coronavirus HKU1	3	2	1	1	1	0	1
Coronavirus NL63	11	4	5^2^	3	0	4	4
Coronavirus OC43	4	3	0	0	2	1	1
hMPV	19	9	1^1^	10	1	3	5
Rhino-/Enterovirus	63	40	14^9^	33	10	9	10
Influenza A virus /-	4	1	1	2	0	1	1
Influenza A virus /H3N2	36	3	20	2	2	11	21
Influenza A virus /H1N1 pmd 2009	35	2	18^1^	8	1	18	8
Influenza B virus	51	4	25^1^_1_	3	9	15	24
PIV 1	2	1	0	2	0	0	0
PIV 2	1	0	1	1	0	0	0
PIV 3	8	3	1	4	1	3	0
PIV *4*	3	3	1	1	2	0	0
RSV	98	34	27^15^	71	5	7	9
*B*. *pertussis*	5	2	5^5^	3	2	0	0
*C*. *pneumoniae*	7	0	3^3^	4	2	1	0
*M*. *pneumoniae*	22	7	7	2	5	10	5
Total	415^a^	147	137^41^_1_	174	54	90	90

^a^No age information was available for seven pathogen results. Adenovirus equals human mastadenovirus A-G, bocavirus equals primate bocaparvovirus 1+2, PIV 1+3 equals human respirovirus 1+3, PIV 2+4 equals human orthorubulavirus 2+4, RSV equals human orthopneumovirus.

Multiple pathogens were detected in 19.5% of the positive samples (65/333), and the highest number of pathogens detected in a single sample was five (human respirovirus 3, human orthorubulavirus 4, human coronavirus OC43, rhino-/enterovirus and primate bocaparvovirus 1+2). The majority of multiple infection samples contained two pathogens, which was the case for 53/65 samples (81.5%). The QIAstat RP detected a bacterial pathogen in 7.6% of all samples (34/445). In 25 out of 34 samples only bacteria were detected and in the remaining nine samples, additional viral pathogens were detected (four samples with two pathogens, four samples with three pathogens and one sample with four pathogens).

The number of each potential pathogen detected by the QIAstat RP in co-infections, by hospitalization and by age group is presented in Table 2. The most frequently detected pathogen was human orthopneumovirus, which was detected in 98 samples, 71 of which (72.4%) came from patients less than 6 years of age. The second most common pathogen was influenza A virus, which was detected in 75 samples, equally distributed between the H1 pdm2009 and H3N2 strains. Rhino-/enterovirus was detected in 63 samples and influenza B virus was detected in 51 samples, highlighting the very active influenza B virus season in early 2018.

### QIAstat RP performance

For 440 of 445 specimens (98.9%), a valid result was obtained by the initial QIAstat RP testing. Five samples (1.1%) were invalid after the first QIAstat RP testing, comprising one run aborted by the analyzer (0.22%), three runs with software errors (0.67%), and one run in which the internal control failed to amplify (0.22%). All five samples were retested with the QIAstat RP and yielded a valid result after the second QIAstat RP testing.

The performance characteristics for individual QIAstat RP targets before discrepancy resolution are presented in Table 3 and the performance characteristics after discrepancy resolution are presented in Table 4.

**Table 3:** Performance summary and characteristics of the QIAstat RP versus the FilmArray RP before resolution of discordant results by Allplex RP.

QIAstat RP compared to FilmArray RP					Confirmation by Allplex			
Pathogen	+/+	+/-	-/+	-/-	++/-	+/--	--/+	-/++
Adenovirus	27	9	5	404	6	3	4	1
Bocavirus^c^	3^c^	2^c^	0^c^	440^c^	NA	NA	NA	NA
Coronavirus 229E	2	0	0	443				
Coronavirus OC43	4	0	0	441				
Coronavirus HKU1^b^	1	2	0	442	NA^b^	NA^b^	NA	NA
Coronavirus NL63	11	0	0	434				
Rhino-/Enterovirus	49	14	8	374	3	11	5	3
hMPV	19	0	2	424			1	1
Influenza virus A /-	2	2^a^	1	440	1		0	1
Influenza A /H3N2	34	2	1	408	2	0	1	0
Influenza A /H1N1	0	0	0	445				
Influenza A /H1-2009 strain (pandemic)	33	2	0	410	2	0		
Influenza virus B	50	1	0	394	1	0		
PIV 1	2	0	0	443				
PIV 2	1	0	0	444				
PIV 3	8	0	1	436			1	0
PIV 4	2	1	0	442	0	1		
RSV	95	3	4	343	3	0	2	2
*B*. *pertussis*	5	0	0	440				
*C*. *pneumoniae*	7	0	0	438				
*M*. *pneumoniae*	21	1	0	423	0	1		
*L*. *pneumophila*	NA	NA	NA	NA	NA	NA	NA	NA
Overall	376	39	22	8908	18	16	14	8

^a^One sample with insufficient volume for discrepancy testing.

^b^Not included in the Allplex RP.

^c^Not included in the FilmArray RP. NA not available.

**Table 4:** Performance summary and characteristics of the QIAstat RP versus the FilmArray RP after resolution of discordant results by the Allplex RP.

QIAstat RP compared to FilmArray RP after Allplex RP discrepancy testing								
Pathogen	TP	FP	FN	TN	Sensitivity	95% CI	Specificity	95% CI
Adenovirus	33	3	1	408	97.1	85.1–99.5	99.3	97.9–99.8
Coronavirus 229E	2	0	0	443	100.0	34.2–100.0	100.0	99.1–100.0
Coronavirus OC43	4	0	0	441	100.0	51.0–100.0	100.0	99.1–100.0
Coronavirus NL63	11	0	0	434	100.0	74.1–100.0	100.0	99.1–100.0
Rhino-/Enterovirus	52	11	3	379	94.4	85.1–98.1	97.2	95.0–98.4
hMPV	19	0	1	425	95.0	76.4–99.1	100.0	99.1–100.0
Influenza A virus /-	3	1^a^	1	440	75.0	30.1–95.4	99.8	98.7–100.0
Influenza A virus /H3N2	36	0	0	409	100.0	90.8–100.0	100.0	99.1–100.0
Influenza A virus /H1N1	0	0	0	445	NA	NA	100.0	99.1–100.0
Influenza A virus /H1-2009 strain (pandemic)	35	0	0	410	100.0	89.8–100.0	100.0	99.1–100.0
Influenza B virus	51	0	0	394	100.0	93.0–100.0	100.0	99.0–100.0
PIV 1	2	0	0	443	100.0	34.2–100.0	100.0	99.1–100.0
PIV 2	1	0	0	444	100.0	20.7–100.0	100.0	99.1–100.0
PIV 3	8	0	0	437	100.0	67.6–100.0	100.0	99.1–100.0
PIV 4	2	1	0	442	100.0	34.2–100.0	99.8	98.7–100.0
RSV	98	0	2	345	98.0	93.0–99.4	100.0	98.9–100.0
*B*. *pertussis*	5	0	0	440	100.0	56.6–100.0	100.0	99.1–100.0
*C*. *pneumoniae*	7	0	0	438	100.0	64.6–100.0	100.0	99.1–100.0
*M*. *pneumoniae*	21	1	0	423	100.0	84.5–100.0	99.8	98.7–100.0
*L*. *pneumophila*	NA	NA	NA	NA	NA	NA	NA	NA
QIAstat RP versus FilmArray RP (human coronavirus) or Allplex RP (bocavirus). No discrepancy testing								
Pathogen	+/+	+/-	-/+	-/-	PPA	95% CI	NPA	95% CI
Bocavirus	3	2	0	440	100.0	43.8–100.0	99.5	98.4–99.9
Coronavirus HKU1	1	2	0	442	100.0	20.7–100.0	99.5	98.4–99.9
Overall	394	21	8	8922	98.0	96.0–99.1	99.8	99.6–99.9

TP is true positive QIAstat RP results, FP is false positive, FN is false negative and TN is true negative results.

^a^Insufficient volume for discrepancy testing. Adenovirus equals human mastadenovirus A-G, bocavirus equals primate bocaparvovirus 1+2, PIV 1+3 equals human respirovirus 1+3, PIV 2+4 equals human orthorubulavirus 2+4, RSV equals human orthopneumovirus.

Before resolution by discrepancy testing, QIAstat RP and FilmArray RP agreed on the detection of 376 pathogens in the 445 samples (Table 3). For all pathogens except human mastadenovirus A-G, rhino-/enterovirus, hMPV, influenza A virus no subtype and human respirovirus 3, the agreement was excellent (Table 3). For human respirovirus 3 and influenza A virus no subtype the total number of positive samples by at least one method was only nine and five respectively. For human mastadenovirus A-G the QIAstat RP and FilmArray RP agreed on 27 positive samples out of 41 initial positive samples (Table 3). After discrepancy testing the sensitivity for the QIAstat RP was 97.1% (33/34, Table 4). For rhino-/enterovirus the QIAstat RP and FilmArray RP agreed on 49 positive samples out of the 71 initial positive samples. After discrepancy testing the sensitivity for the QIAstat RP was 94.4% (52/55). For hMPV the QIAstat RP and FilmArray RP agreed on 19 positive samples out of 21 initial positive samples. After discrepancy testing the sensitivity was 95.0% (19/20).

After resolution by discrepancy testing on the Allplex RP, a total of 402 pathogen results were considered as true positive (Table 4), of which QIAstat RP detected 394, for an overall PPA of 98.0% (95%CI 96.0%-99.1%). After discrepancy testing, the overall NPA of the QIAstat RP was 99.8% (95%CI 99.6%-99.9).

Twenty-two pathogens detected by FilmArray RP were not detected by QIAstat RP (Table 3). Fourteen of these were also not detected by AllPlex RP and were considered as true negatives, leaving 8 pathogen detections as false negatives (Table 4). Thirty-nine pathogens detected by QIAstat RP were not detected by FilmArray RP (Table 3). Twenty-one of these were also not detected by AllPlex RP and were considered as false positives (Table 4).

Three of the 22 pathogens detected by FilmArray RP but not detected by QIAstat RP were detected in single infection samples and additional two of the pathogens not detected by QIAstat RP were both detected in the same double infection sample. The five pathogens involved were rhino-/enterovirus (three), human mastadenovirus A-G (one) and human orthopneumovirus (one). All off these five pathogens were detected by the discrepancy method (Allplex RP). The remaining 17 pathogens not detected by QIAstat RP but detected by FilmArray RP were from 17 samples with two or more pathogens. Three of these 17 pathogens (one hMPV, one influenza A virus, no subtype and one human orthopneumovirus) were detected by the discrepancy method adding up to a total of 8 false negative pathogens.

QIAstat RP detected 39 pathogens not detected by FilmArray RP. 10 of these pathogens were in single infection samples and the detected pathogens were rhino-/enterovirus (n = 1, CT value 27.6), influenza B virus (n = 1, CT value 20.7), influenza A virus, no subtype (n = 1, CT value 34.2), influenza A virus subtype H3N2 (n = 2, CT values 32.7 and 33.7), human orthopneumovirus (n = 2, CT values 28.8 and 32.9) and human mastadenovirus A-G (n = 3, CT values 21.3, 22.4 and 22.7). The influenza A virus with no detected subtype sample could not be discrepancy tested due to insufficient residual volume and was accordingly considered false positive by the QIAstat RP. One additional single-infection pathogen (the rhino-/enterovirus) was considered false positive, as this result could not be confirmed positive by discrepancy testing. The remaining eight single-infection pathogens were confirmed positive by the discrepancy method and thus considered true positives (Table 4). The remaining 29 pathogens detected by QIAstat RP but not detected by FilmArray RP were from samples with two or more pathogens. Nineteen of these 29 pathogens (ten rhino-/enterovirus, CT values 26.9, 30.6, 32.2, 32.5, 32.6, 33.1, 33.1, 33.3, 33.5 and 34.4, three human mastadenovirus A-G, CT values 33.4, 33.7 and 37.3, one human orthorubulavirus 4, CT value 33.6, one *Mycoplasma pneumoniae*, CT value 33.3, two primate bocaparvovirus 1+2, CT values 34.1 and 34.9 and two human coronavirus HKU1, CT values 31.7 and 33.1) could not be confirmed by discrepancy testing and were thus considered false positives, for a total of 21 false positive pathogens (Table 4). The remaining 10 pathogens were confirmed by the Allplex RP from samples with two or more pathogens (three human mastadenovirus A-G, CT values 30.5, 30.8 and 33.3, three rhino-/enterovirus, CT values 29.7, 30.0 and 32.2, human orthopneumovirus, CT value 16.2, two influenza A virus subtype H3N2 and one influenza A virus, no subtype with no available CT value).

It was not possible to assess the sensitivity of the QIAstat RP for *Legionella pneumophila* or influenza A virus subtype H1N1 as these organisms were not detected by any of the methods. Primate bocaparvovirus 1+2 was detected by the QIAstat RP in five specimens, all containing at least one other potential pathogen. The Allplex RP confirmed the detected primate bocaparvovirus 1+2 in three of five specimens. Because primate bocaparvovirus 1+2 is not included in the panel of the reference method, the results are listed separately and are expressed as PPA/NPA.

## Discussion

This study demonstrates for the first time the clinical performance of the new QIAstat RP assay for detection of 21 respiratory pathogens. By comparison to the FilmArray RP, the overall PPA and NPA after discrepancy testing were 98.0% (95% CI: 96.0%-99.1%) and 99.8% (95% CI: 99.6%-99.8) respectively. In general, the QIAstat RP assay showed excellent agreement with FilmArray RP. For all pathogens included on the QIAstat RP test menu, except rhino-/enterovirus (52 TP and 379 TN samples), influenza A virus, no subtype (3 TP and 440 TN samples) and hMPV (19 TP and 425 TN samples), the QIAstat RP showed sensitivity/PPA >97% and specificity/NPA >99% (Table 4). The reason for the discordant results for rhino-/enterovirus is not known but may potentially be explained by different diagnostic tests targeting different genetic regions of the rhino/enterovirus genome.

The CT values for the pathogen targets present in the discrepant QIAstat positive RP and FilmArray RP negative samples were detected by QIAstat with CT values in the range 30–37, which may be considered as weakly positive. Only three targets were detected as QIAstat RP positive and FilmArray RP negative target with CT values below 30, one human orthopneumovirus (CT 16.2) and two rhino-/enterovirus (CT 26.9 and 29.7) and thus as strong positive samples.

It would have been beneficial to resolve discrepancy results by sequencing as this potentially would have clarified the discordant results for rhino-/enterovirus. This was not possible as both the QIAstat RP and FilmArray RP as assays are performed in sealed samples-to-answer cartridges that are constructed to prevent access to sample material remaining in the cartridge.

The QIAstat RP assay requires one manual pipetting step to load the patient sample into test cartridge. The test cartridge ID and patient/sample ID is entered by on-board scanner and the total assay time is approximately 69 minutes. The QIAstat Dx DiagCORE analyzer offers a small footprint, traceable internal inhibition controls (by amplification curve as well as Ct value), low maintenance requirements and low failure rate as well *as* seamless connectivity and integration with hospital and laboratory information systems. In addition, the QIAstat Dx DiagCORE analyzer offers access to amplification curves and Ct values of all detected pathogen targets. The QIAstat RP assay cartridge has on-board wet and dry reagents, build-in amplification inhibition controls, detects 21 pathogens in a total of 8 separate reaction chambers, offers a direct dry swab testing option via a separate cartridge input port and stores at room temperature. The QIAstat RP is CE IVD marked and FDA approval is pending.

The syndromic diagnostic approach for ARTI may provide multiple benefits for the institution (e.g. prudent use of side room isolation facilities, reduced prescription of antimicrobials, targeted use of antivirals) The benefits have been partly described in previous publications. Trabattoni *et al* reported a significant reduction in Emergency Department (ED) length of stay as well as a significant reduction in hospitalization rate for patients tested by the Alere i influenza assay in the ED [22], and Hansen *et al* reported a positive impact on admittance rate as well as total cost by Roche Liat influenza testing in the ED setting [14]. Brendish *et al* reported on encouraging findings of PoC testing of respiratory viruses, as their studies showed that this testing was associated with a reduced length of hospital stay, resulted in more single doses or brief courses of antibiotics as well as in an improved influenza detection and antiviral use [15]. However, understanding of the full impact on patient management by the syndromic testing approach for ARTI as well as the cost-benefit will require more prospective outcome studies. Such studies would also provide information regarding which patient population(s) will benefit the most from respiratory tract syndromic testing and what the added benefits of syndromic testing compared to selected target testing (e.g. influenza A/B virus, human orthopneumovirus, hMPV, *Bordetella pertussis* or combinations hereof).

This study has several limitations. First, as this study was not designed as an epidemiological study, the included clinical samples were not collected consecutively and the detected co-infection rates and the frequency of different pathogens in the co-infected samples may not reflect the actual co-infection rates in the patient population at the time of sample collection. Second, as several pathogens, e.g. coronaviruses, *B*. *pertussis*, *C*. *pneumoniae* and parainfluenzavirus 1–4, were only detected in few clinical samples, the performance of the QIAstat RP assay for detection of these pathogens is uncertain.

In conclusion, in this first clinical study of the new QIAstat RP assay performed in two diagnostic laboratories providing 445 clinical samples for analysis, we observed excellent diagnostic accuracy of the QIAstat RP assay compared to the comparator (FilmArray RP). The QIAstat RP assay could potentially impact positively on antimicrobial stewardship, hospital admittance and use of side room contact isolation facilities.

## References

[1] GBD 2017 Influenza Collaborators. Mortality, morbidity, and hospitalisations due to influenza lower respiratory tract infections, 2017: an analysis for the Global Burden of Disease Study 2017. Lancet Respir Med. 2019; 7: 69–89. 10.1016/S2213-2600(18)30496-X 30553848 PMC6302221

[2] Brealey JC, Sly PD, Young PR, Chappell KJ. Viral bacterial co-infection of the respiratory tract during early childhood. FEMS Microbiol Lett. 2015;362: 1–11. 10.1093/femsle/fnv062 25877546

[3] Lambkin-Williams R, Noulin N, Mann A, Catchpole A, Gilbert AS. The human viral challenge model: Accelerating the evaluation of respiratory antivirals, vaccines and novel diagnostics. Respir Res. 2018;19: 123 10.1186/s12931-018-0784-1 29929556 PMC6013893

[4] Fleming DM, Taylor RJ, Lustig RL, Schuck-Paim C, Haguinet F, Webb DJ, et al Modelling estimates of the burden of respiratory syncytial virus infection in adults and the elderly in the United Kingdom. BMC Infect Dis. 2015;15: 443 10.1186/s12879-015-1218-z 26497750 PMC4618996

[5] Choi S-H, Hong S-B, Ko G-B, Lee Y, Park HJ, Park S-Y, et al Viral infection in patients with severe pneumonia requiring intensive care unit admission. Am J Respir Crit Care Med. 2012;186: 325–332. 10.1164/rccm.201112-2240OC 22700859

[6] Hong HL, Hong SB, Ko GB, Huh JW, Sung H, Do KH, et al Viral infection is not uncommon in adult patients with severe hospital-acquired pneumonia. PLoS One. 2014;9: e95865 10.1371/journal.pone.0095865 24752070 PMC3994115

[7] Loubet P, Voiriot G, Houhou-Fidouh N, Neuville M, Bouadma L, Lescure FX, et al Impact of respiratory viruses in hospital-acquired pneumonia in the intensive care unit: A single-center retrospective study. J Clin Virol. 2017;91: 52–57. 10.1016/j.jcv.2017.04.001 28494435 PMC7106511

[8] Visseaux B, Burdet C, Voiriot G, Lescure FX, Chougar T, Brugière O, et al Prevalence of respiratory viruses among adults, by season, age, respiratory tract region and type of medical unit in Paris, France, from 2011 to 2016. PLoS One. 2017;12: e0180888 10.1371/journal.pone.0180888 28708843 PMC5510824

[9] Voiriot G, Visseaux B, Cohen J, Nguyen LBL, Neuville M, Morbieu C, et al Viral-bacterial coinfection affects the presentation and alters the prognosis of severe community-acquired pneumonia. Crit Care. 2016;20: 375 10.1186/s13054-016-1517-9 27852281 PMC5112669

[10] CDC. 2018. Prevention strategies for seasonal influenza in healthcare settings. https://www.cdc.gov/flu/professionals/infectioncontrol/healthcaresettings.htm

[11] Li J, Song X, Yang T, Chen Y, Gong Y, Yin X, et al Systematic review of antibiotic prescription associated with upper respiratory tract infections in China. Med (United States). 2016;95: e3587. 10.1097/MD.0000000000003587 PMC4902500 27175658

[12] Fleming-Dutra KE, Hersh AL, Shapiro DJ, Bartoces M, Enns EA, File TM, et al Prevalence of inappropriate antibiotic prescriptions among US ambulatory care visits, 2010–2011. JAMA—J Am Med Assoc. 2016;315: 1864–1873. 10.1001/jama.2016.4151 27139059

[13] Schreckenberger PC, McAdam AJ. Point-counterpoint: Large multiplex PCR panels should be first-line tests for detection of respiratory and intestinal pathogens. J Clin Microbiol. 2015;53: 3110–3115. 10.1128/JCM.00382-15 25762770 PMC4572537

[14] Hansen GT, Moore J, Herding E, Gooch T, Hirigoyen D, Hanson K, et al Clinical decision making in the emergency department setting using rapid PCR: Results of the CLADE study group. J Clin Virol. 2018;102: 42–49. 10.1016/j.jcv.2018.02.013 29494950 PMC7106512

[15] Brendish NJ, Malachira AK, Armstrong L, Houghton R, Aitken S, Nyimbili E, et al Routine molecular point-of-care testing for respiratory viruses in adults presenting to hospital with acute respiratory illness (ResPOC): a pragmatic, open-label, randomised controlled trial. Lancet Respir Med. 2017;5: 401–411. 10.1016/S2213-2600(17)30120-0 28392237 PMC7164815

[16] Rappo U, Schuetz AN, Jenkins SG, Calfee DP, Walsh TJ, Wells MT, et al Impact of early detection of respiratory viruses by multiplex PCR assay on clinical outcomes in adult patients. J Clin Microbiol. 2016;54: 2096–2103. 10.1128/JCM.00549-16 27225406 PMC4963510

[17] Rogers BB, Shankar P, Jerris RC, Kotzbauer D, Anderson EJ, Watson JR, et al Impact of a rapid respiratory panel test on patient outcomes. Arch Pathol Lab Med. 2015;139: 636–641. 10.5858/arpa.2014-0257-OA 25152311

[18] Brendish NJ, Malachira AK, Beard KR, Ewings S, Clark TW. Impact of turnaround time on outcome with point-of-care testing for respiratory viruses: A post hoc analysis from a randomised controlled trial. Eur Respir J. 2018;52: 1800555 10.1183/13993003.00555-2018 29946003

[19] Landes MB, Neil RB, McCool SS, Mason BP, Woron AM, Garman RL, et al The frequency and seasonality of influenza and other respiratory viruses in Tennessee: two influenza seasons of surveillance data, 2010–2012. Influenza Other Respi Viruses. 2013;7: 1122–1127. 10.1111/irv.12145 PMC4634273 23962104

[20] Marinović AB, Swaan C, Van Steenbergen J, Kretzschmar M. Quantifying reporting timeliness to improve outbreak control. Emerg infect dis. 2015;21: 209–216. 10.3201/eid2102.130504 25625374 PMC4313625

[21] Uyeki TM, Bernstein HH, Bradley JS, Englund JA, File TM, Fry AM, et al Clinical practice guidelines by the infectious diseases society of America: 2018 update on diagnosis, treatment, chemoprophylaxis, and institutional outbreak management of seasonal influenza. Clin Infect Dis. 2018 10.1093/cid/ciy866 30566567 PMC6653685

[22] Trabattoni E, Le V, Pilmis B, Pean de Ponfilly G, Caisso C, Couzigou C, et al Implementation of Alere i influenza A & B point of care test for the diagnosis of influenza in an ED. Am J Emerg Med. 2018;36: 916–921. 10.1016/j.ajem.2017.10.046 29137903

